# The Association between Health-Enhancing Physical Activity and Quality of Life in Patients with Chronic Kidney Disease: Propensity Score Matching Analysis

**DOI:** 10.3390/ijerph19031318

**Published:** 2022-01-25

**Authors:** Tae Ryom Oh, Hong Sang Choi, Sang Heon Suh, Chang Seong Kim, Eun Hui Bae, Suah Sung, Seung Hyeok Han, Kook Hwan Oh, Seong Kwon Ma, Soo Wan Kim

**Affiliations:** 1 Department of Internal Medicine, Chonnam National University Hospital, 42 Jebongro, Gwangju 61469, Korea; tryeomoh@hanmail.net (T.R.O.); hongsang38@hanmail.net (H.S.C.); medssh1984@gmail.com (S.H.S.); laminion@hanmail.net (C.S.K.); baedak76@gmail.com (E.H.B.); 2 Department of Internal Medicine, Chonnam National University Medical School, 42 Jebongro, Gwangju 61469, Korea; 3 Department of Internal Medicine, Eulji Medical Center, Eulji University, Seoul 01830, Korea; soi@eulji.ac.kr; 4 Department of Internal Medicine, College of Medicine, Institute of Kidney Disease Research, Yonsei University, Seoul 03722, Korea; hansh@yuhs.ac; 5 Department of Internal Medicine, Seoul National University, Seoul 03080, Korea; ohchris@hanmail.net

**Keywords:** chronic kidney disease, physical activity, quality of life, exercise, life

## Abstract

We investigate the association between health-enhancing physical activity and the quality of life in patients with non-dialysis chronic kidney disease. We performed data analysis on 1618 of 2238 patients from 2011 to 2016, obtained from the KoreaN Cohort Study for Outcome in Patients with Chronic Kidney Disease (KNOW-CKD). Health-related quality of life was measured using the Korean version 1.3 of Kidney Disease Quality of Life short-form questionnaire. Health-enhancing physical activity was defined as 150 min of moderate-intensity or 75 min of vigorous-intensity aerobic physical activity throughout the week. Propensity score matching analysis and linear regression was performed to estimate the effect of health-enhancing physical activity on health-related quality of life. The estimate of average treatment effects was 2.60 in the kidney component summary score, 4.45 in the physical component summary score, and 4.24 in the mental component summary score. In all component summary scores and most of their subscales, health-enhancing physical activity showed a significant association with health-related quality of life. Subgroup and sensitivity analyses also showed robust results. This study suggests that health-enhancing physical activity elevated quality of life in patients with non-dialysis chronic kidney disease. The results can contribute to encourage physical activity in patients with chronic kidney disease.

## 1. Introduction

Chronic kidney disease (CKD) is an emerging public health problem globally [1]. It is well known that CKD patients have poor health-related quality of life (HRQoL) compared to that of the general population [2]. Progression of CKD to end-stage renal disease results in a significant decrease in the quality of life (QoL) of the patients [2,3]. The World Health Organization (WHO) defines the QoL as ‘a person’s individual perception of one’s status in life with respect to one’s goal, expectations, norms, and interests within the context of the culture and value system in which he lives’ [4]. As the prevalence of chronic diseases and life expectancy increases, QoL is recognized as an important factor in the medical field. Since QoL has been in the spotlight, many researchers have studied the association between QoL and the patients’ prognoses. In many studies, QoL is not only a surrogate marker of chronic disease progression but also a risk factor of various outcomes, including mortality [5], and cardiovascular [6], and renal outcomes [7].

The World Health Organization (WHO) has recommended guidelines for physical activity for an individual’s healthy life and better QoL [8,9]. Health-enhancing physical activity (HEPA), which is recommended by WHO, has been recommended to maintain the health of the general population [9]. HEPA has various beneficial effects on individual health, including improved QoL, cognitive function, and sleep, reducing all-cause and cardiovascular disease mortality, lower risk of diabetes mellitus and hypertension [8]. Although the KDIGO guidelines recommend physical activity in CKD patients, the evidence for physical activity in the CKD population is limited and uncertain (Grade 2C) [10]. The association between HEPA and QoL in patients with CKD is still unclear. Considering the relationship between QoL and the prognosis of patients with CKD and previous studies that HEPA improves QoL in the general population, analysis of the association between HEPA and QoL in patients with CKD is an important task. We hypothesized that HEPA would improve HRQoL of the CKD patients. The purpose of this study was to investigate the association between HEPA and HRQoL in patients with non-dialysis CKD.

## 2. Materials and Methods

### 2.1. Study Population

**Figure 1:**
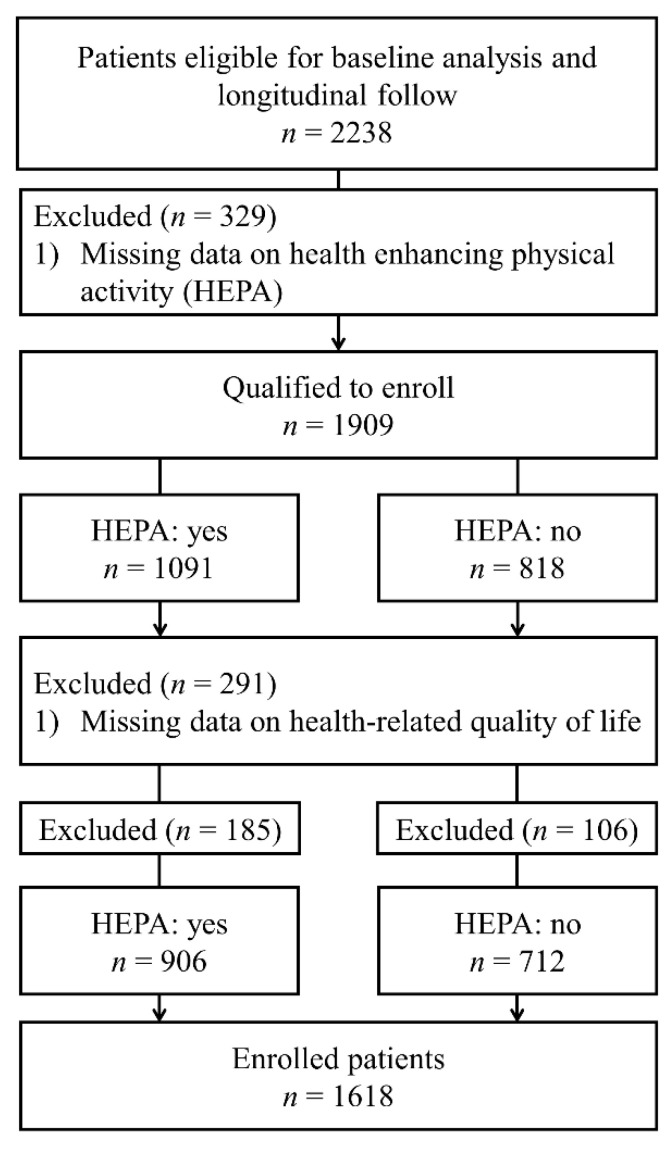
Flow chart for enrollment of the study population. Flow diagram for patient’s enrollment.

The KoreaN cohort study for Outcome in patients With Chronic Kidney Disease (KNOW-CKD) is a nationwide prospective cohort study in Korea which includes patients with non-dialysis CKD from stages 1-5 (NCT01630486 at http://www.clinicaltrials.gov, accessed on 6 December 2021). A total of 2238 patients were enrolled between 2011 and 2016 from a tertiary care hospital in Korea. Patients with heart failure (New York Heart Association functional class 3 or 4); or liver cirrhosis (Child-Pugh class 2 or 3); or previous maintenance dialysis or organ transplantation; or inability or unwillingness to provide written consent; or history of current malignancy; or pregnancy; or a single kidney were excluded at enrollment. The detailed study design, methods, and protocols have been previously described [11]. The study was conducted in accordance with the principles of the Declaration of Helsinki, and the study protocol was approved by the institutional review board of each of the eight participating clinical centers. Written informed consent was obtained from all patients at each center before enrollment. Among the total patients included in KNOW-CKD, 620 patients were excluded due to the missing values or unclear response to the questionnaire about the HRQoL, HEPA, and bone mineral density test. Therefore, this study included 1618 patients (Figure 1).

### 2.2. Data Collection and Measurements

Baseline demographic details and clinical data of the entire study population, including age, sex, smoking history, cause of CKD, economic status, educational level, comorbidities, and medications were surveyed by a well-trained research coordinator. Height, weight, and circumferences of waist and hip were also measured. BMI was calculated by dividing the initial body weight (kg) by height squared (m^2^). Blood pressure was measured using an electronic sphygmomanometer in the clinic after 5 min of seated rest. In addition, 10 mL of a blood sample, first-voided urine sample, and a 24-h urine was collected from all the patients for biochemical analyses. The collected samples were sent to the central laboratory (Lab Genomics, Seongnam-si, Korea) for measurement of the complete blood count and blood chemistries. Serum creatinine level was measured by the isotope dilution mass spectroscopy (IDMS)-traceable method.

### 2.3. Health-Related Quality of Life

The Korean version 1.3 of Kidney Disease Quality of Life short form (KDQOL-SF) was used to evaluate HRQoL. The KDQOL-SF comprises a kidney disease component summary (KDCS) and the Medical Outcome Study Short Form-36 Health Survey (SF-36) including a physical component summary (PCS) and a mental component summary (MCS). KDCS is an arithmetic mean of kidney disease-targeted scales which includes 11 subscales: (1) symptom/problems, (2) work status, (3) sexual function, (4) physician encouragement, (5) effects of kidney disease, (6) cognitive function, (7) sleep, (8) patient satisfaction, (9) burden of kidney disease, (10) quality of social interaction, (11) social support. The PCS and MCS each contain four subscales: (1) physical function, (2) role physical, (3) bodily pain, (4) general health, (5) emotional well-being, (6) role-emotional, (7) social function, and (8) energy/fatigue. The former four subscales are summarized in the PCS, and the latter four subscales are summarized in the MCS. Responses to each question of the questionnaire were converted to equivalent scores, and each scale ranged from 0 to 100, with higher numerical scores indicating better HRQoL in that subscale.

### 2.4. Variables

Estimated glomerular filtration rate (eGFR) was calculated using the four-variable Chronic Kidney Disease Epidemiology Collaboration (CKD-EPI) equation [12]. HEPA was defined as 150 min of moderate-intensity or 75 min of vigorous-intensity aerobic physical activity throughout the week. The amount of physical activity was calculated based on a self-reported questionnaire. A National Health and Nutrition Survey questionnaire of Korea was modified for the patients with CKD to survey the physical activity of the patients. Diabetes mellitus was defined as serum hemoglobin A1c ≥ 6.5%, fasting glucose ≥126 mg/dL, or a previous diagnosis of diabetes. A monthly family income of below ￦1,500,000 (approximately United States $1500) was defined as low income, ￦1,500,000–4,500,000 as middle income, and above ￦4,500,000 to high income. Education status was classified as an academic background of less than elementary school graduation, middle school graduation, high school graduation, and above university graduation. Marital status was defined as (1) married, (2) unmarried and (3) divorced or widowed.

### 2.5. Statistical Analysis

A student’s *t*-test for normally distributed data and the Mann–Whitney U test for non-normal distribution data were used to compare the clinical characteristics between the groups. The Chi-square test and Cochran–Armitage trend test were used to compare the categorical variables (two groups and more than two groups, respectively). We applied a simple imputation method for missing data using the ‘MICE’ package [13], because of the low percentage of missing values (Table S1). The propensity scores were estimated by using logistic regression for propensity score matching analysis, and we used the full matching method using the ‘MatchIt’ package [14]. The standardized mean difference (<0.1) was reviewed before and after matching to assess the optimal balance of covariates. We calculated the estimates and confidence intervals of the treatment effect of HEPA with non-parametric bootstrapping using the ‘Zelig’ package [15] and compared the beta coefficients and confidence intervals (CI) from adjusted linear regression with ordinary least squares (OLS). We also performed the subgroup and sensitivity analysis to investigate the robustness of this study and assessed the impact of omitted variable bias. Data were analyzed and plotted using R language (version 4.0.2; The R Foundation for Statistical Computing, Vienna, Austria) [16]. All statistical tests were two-tailed, and *p*-values < 0.05 were considered statistically significant.

## 3. Results

### 3.1. Clinical Characteristics of the Study Participants

Among a total of 1618 patients, the mean age was 52.3 years, and the number of females was 605 (37.4%). The median baseline eGFR was 48.0 mL/min/1.73 m^2^ and 509 (31.5%) patients had diabetes mellitus. Patients with higher education (above high school) were 1287 (79.6%), and most patients (82.8%) were married. A high prevalence of hypertension (95.7%) in this study population was observed. We compared the clinical characteristics between HEPA (patients who performed HEPA) and non-HEPA (patients who did not perform HEPA) groups, and significant differences in clinical characteristics were observed between the two groups. The HEPA group had a higher proportion of males and higher employment status than the non-HEPA group. The ratio of divorced or widowed subjects was higher in the non-HEPA group. In addition, a higher level of eGFR and a lower proportion of current smokers was observed in the HEPA group. The difference in the level of hemoglobin between the two groups was statistically significant; however, there was no difference in clinical practice. Age, body mass index, waist-hip ratio, serum albumin, serum uric acid, fasting glucose, C-reactive protein, protein to creatinine ratio, high-density lipoprotein, triglycerides, mean ankle-brachial pressure index, and bone mineral density of the total spine did not show any statistically significant difference between the groups. All component summary scores of HRQoL was higher in the HEPA group than in the non-HEPA group. The detailed data are summarized in Table 1. Data prior to imputation for missing values are also described in Table S1.

### 3.2. Linear Regression with Ordinary Least Squares Method

We constructed linear regression models to estimate the beta coefficients of HEPA on HRQoL, and the covariates were selected based on the outcomes of the univariate analysis and clinical significance. HEPA showed a statistically significant effect on all component summary scores even after adjusting for independent variables that could affect the quality of life. Beta coefficients (CI) of HEPA were 2.560 (1.496–3.624) for KDCS, 4.058 (2.517–5.599) for PCS, and 4.193 (2.529–5.857) for MCS in the fully adjusted linear regression model. Most subscales including symptoms/problem lists, sexual function, effects of kidney disease, sleep, the burden of kidney disease, quality of social interaction, social support, physical function, role-physical, bodily pain, general health, emotional well-being, role-emotional, social function, and energy/fatigue were significantly associated with HEPA in linear regression. The most powerful effects of HEPA on the subscales of the component summary scores were on sexual function (beta coefficients, 6.577; CI, 2.526–10.628) in KDCS, general health (beta coefficients, 5.717) in PCS, and energy/fatigue (beta coefficients, 6.62; CI, 4.803–8.437) in MCS. The detailed results were summarized in Table 2 and Table S2.

### 3.3. Propensity Score Matching Analysis

**Figure 2:**
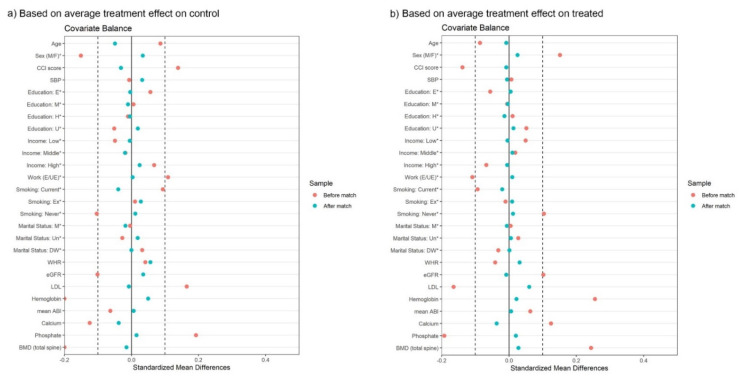
Balance of covariates in propensity score matching. The standardized difference of all covariates was below 0.1. significant at *p*-value < 0.05. (**a**) Based on average treatment effect on control, (**b**) Based on average treatment effect on treated. Abbreviations: E in Education, below elementary school; M in Education, middle school; H in Education, high school; U in Education, above university; E in Work, employed; UE in Work, unemployed; WHR, waist-hip ratio; eGFR, estimated glomerular filtration rate; LDL, low density lipoprotein; ABI, ankle brachial pressure index; BMD, bone mineral density.

To control the influence of confounding variables, we performed propensity score matching analysis. Covariates were selected based on the results of the univariate analysis and the clinical significance. A full matching method was applied to ensure a balance between the covariates, and the covariate balancing was performed based on the average treatment effect on control (ATC) and average treatment effect on treated (ATT). The matching results of covariate balance are visualized in Figure 2, and all covariates have smaller values of the standard mean difference than 0.1, which was the reference value.

**Figure 3:**
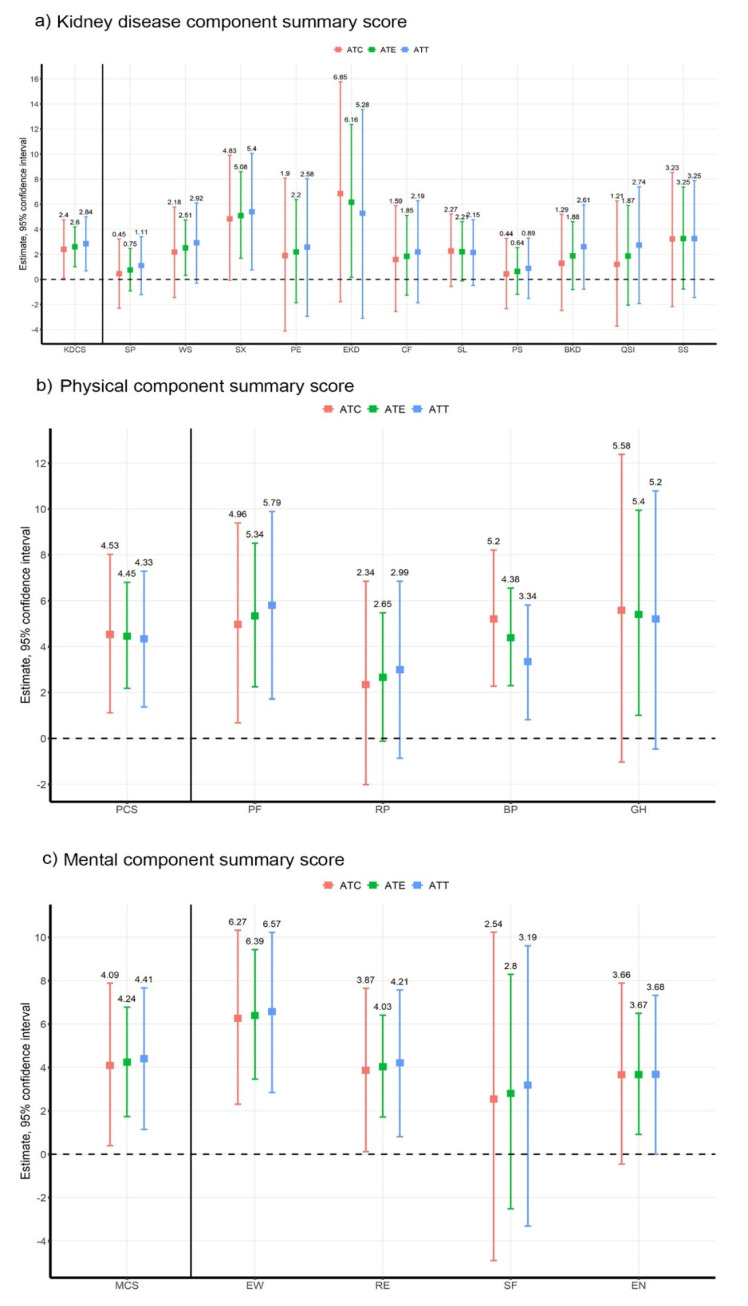
Estimates and confidence interval of treatment effect of health-enhancing physical activity on quality of life. Treatment effect of health-enhancing physical activity on all component summary scores and its subscales. Significance at *p*-value < 0.05. (**a**) Kidney component summary score, (**b**) physical component summary score, (**c**) mental component summary score. Abbreviations: ATC, average treatment effect on control; ATE. average treatment effect; ATT, average treatment effect on treated; KDCS, kidney disease component summary, SP, Symptom/problems; WS, Work status; SF, Sexual function; PE, Physician encouragement; EKD, Effects of kidney disease; CF, Cognitive function; SL, Sleep; PS, Patient satisfaction; BKD, Burden of kidney disease; QSI, Quality of social interaction; SS, Social support; PCS, physical component summary; PF, Physical Function; RP, Role physical; BP, Bodily Pain, GH, General Health; MCS, mental component summary; EW, Emotional well-being; RE, Role-Emotional; SF, Social Function; EN, Energy/Fatigue.

After obtaining covariate balance, we estimated the ATT, and average treatment effect (ATE) with non-parametric bootstrapping techniques (10,000 times). Estimates of the treatment effect of HEPA on all subscales and component summary scores are shown in Figure 3 with CI of beta coefficients of linear regression. The estimates for treatment effect (CI) were 2.60 (1.104–4.188) for ATE, 2.84 (0.685–4.995) for ATT, and 2.40 (0.100–4.764) for ATC in KDCS; 4.45 (2.177–6.799) for ATE, 4.33 (1.372–7.283) for ATT, and 4.53 (1.116–8.02) for ATC in PCS; and 4.24 (1.732–6.782) for ATE, 4.41 (1.140–7.672) for ATT, and 4.09 (0.394–7.884)for ATC in MCS, respectively. The CI of all estimates of treatment effects was above zero, indicating that the estimates were statistically significant. Similar to the results of linear regression, HEPA improved all component summary scores by approximately 2-4 points. However, the association between HEPA and subscales were significantly different from linear regression. Based on ATE, only the KDCS subscales of work status, sexual function, and effect of kidney disease showed statistical significance; however, most subscales of PCS and MCS, including physical function, bodily pain and general health, emotional well-being, role-emotional, and energy/fatigue showed statistically significant results.

We performed a sensitivity analysis to evaluate the robustness of the increase in HRQoL due to HEPA in case of deviation from the conditional independence assumption, which is an important assumption in propensity score matching analysis. The sensitivity analysis showed a *Γ* value of 1.4, at which the statistical significance disappears. It indicates that the improvement in HRQoL by the propensity score matching analysis becomes statistically insignificant when the odds to be assigned to the HEPA group are increased by 140% or more due to unobserved factors. However, these statements assume that the impact of unobserved variables on HRQoL is powerful, which rarely is the case.

### 3.4. Subgroup Analysis

**Figure 4:**
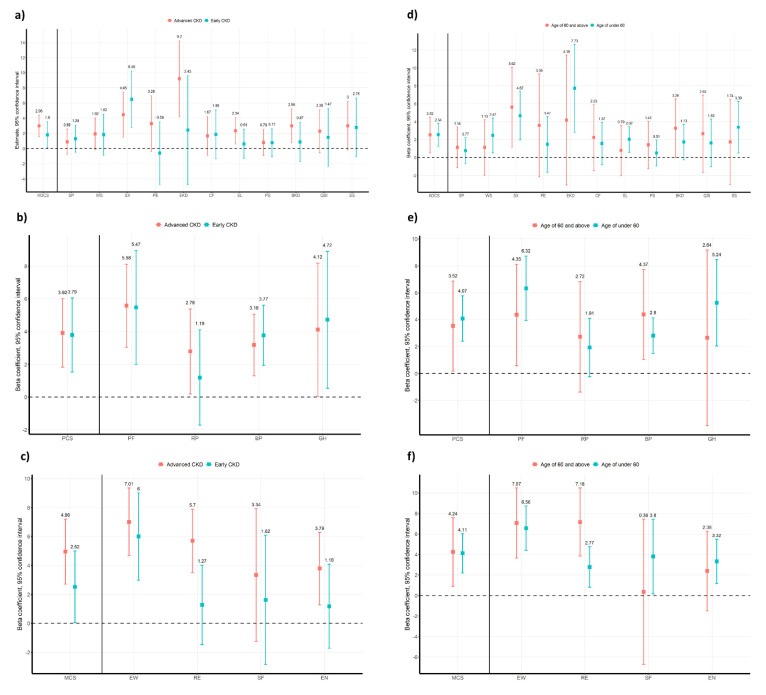
Difference of quality of life by health-enhancing physical activity in subgroups based on renal function and age. The difference of beta coefficients of health-enhancing physical activity on all component summary scores and its subscales in subgroup analyses. Significant at *p*-value < 0.05. (**a**) Kidney component summary score, (**b**) Physical component summary score, (**c**) Mental component summary score with subgroups of CKD and (**d**) Kidney component summary score, (**e**) Physical component summary score, (**f**) Mental component summary score with subgroups of age. Abbreviations: CKD, chronic kidney disease; KDCS, kidney disease component summary, SP, Symptom/problems; WS, Work status; SF, Sexual function; PE, Physician encouragement; EKD, Effects of kidney disease; CF, Cognitive function; SL, Sleep; PS, Patient satisfaction; BKD, Burden of kidney disease; QSI, Quality of social interaction; SS, Social support; PCS, physical component summary; PF, Physical Function; RP, Role physical; BP, Bodily Pain, GH, General Health; MCS, mental component summary; EW, Emotional well-being; RE, Role-Emotional; SF, Social Function; EN, Energy/Fatigue.

Both early and advanced CKD groups showed statistically significant relationships between HEPA and HRQoL (Figure 4a–c) in linear regression with OLS. However, in KDCS and MCS, the improvement of HRQoL in the advanced CKD group was greater than in the early CKD group. Subscales of KDCS except sexual function were more closely related with HEPA in the advanced CKD group compared to the early CKD group. In addition, subscales of MCS (emotional well-being, role-Emotional, and energy/fatigue) had statistically significant relationships with HEPA in the advanced CKD group, whereas only emotional well-being showed a significant result in the early CKD group. In the subgroup analysis based on age, the influence of HEPA on HRQoL was greater in the group under 60 years of age compared to group of 60 and above years of age in the overall PCS domains. Otherwise, the effect of HEPA on MCS domains were greater in the group of 60 and above years of age than group under 60 years of age. The detailed results of subgroup analysis based on age were summarized in Figure 4d–f.

## 4. Discussion

HEPA for patients with non-dialysis CKD was associated with all component summary scores of HRQoL in both linear regression and propensity score matching analysis. Propensity score matching was applied to minimize the effect of confounders, and the sensitivity analysis also showed robust and consistent results. Our study reveals the clinical effectiveness of HEPA in improving the HRQoL of patients with non-dialysis CKD.

HRQoL is the assessment tool for QoL, which focuses on the effect of health status, and its importance in patient care is increasing. Patients with end-stage renal disease have a lower HRQoL score compared to that of the general population [3,17]. KDOQI guidelines [18] recommend repeated measurements of HRQoL to indicate the quality of care in patients on hemodialysis. KDQOL-SF36 is one of the methods that can evaluate the HRQoL in dialysis patients and has the advantage of comprehensively evaluating QoL, including disease burden and physical and mental status. We used KDQOL-SF36 in this study because the efficiency, validity, and reliability of KDQOL-SF36 has been confirmed by several studies [19,20,21]. In addition, HEPA is one of the recommendations by WHO for regular physical activity. Regular physical activity has various health benefits, including decreased overall mortality [22,23] and mortality due to cardiovascular disease [23], hypertension [24], and renal outcomes [25]. Despite its importance, there is little research on the utility and optimal level of regular physical activity in patients with non-dialysis CKD.

Improving HRQOL is an important goal to maintain health. HRQoL is affected by various factors including economic status [26], marital status [27], educational status [28], age [26,29], disease burden [29], and others. These factors interact with each other as confounding variables rather than acting independently of each other. If a confounding variable is included as a covariate in linear regression with OLS, the estimated effects of variables are likely to be biased. The propensity score matching analysis is the best way to evaluate the influence of the variable of interest on the dependent variable in a retrospective observational study by controlling the mutual interaction of the confounding variables [30]. In this study, there is no significant difference between ATT, ATC and ATE of HEPA in propensity score matching analyses. ATT is the average expected effect in the treated group, ATC is the average expected effect in the control group, and ATE is the average expected effect in the entire study population. From the similarity of the above values of three treatment effects, we may cautiously infer that there will be a potential effect of HEPA even on the group without active physical activity. Linear regression with OLS and propensity score matching analysis also showed similar results in terms of component summary scores; however, there were some differences in the subscale scores. There was no significant difference in component summary scores and its subscale scores in linear regression with OLS; however, considerable large differences were seen in the KDCS domain in propensity score matching analysis. This inconsistency might be due to the bias caused by the confounding variables [31]. In addition, recent research reported that component summary scores could not completely reflect its subscales [32]; this supports our inconsistent results between the component summary score and its subscales. In this study, we found that the association of HEPA in the PCS and MCS domains was greater than in the KDCS domain, and we confirmed that our results were robust through sensitivity analysis. HEPA has a significant relationship with the symptomatic burden in CKD. We also found that improvements in HRQoL with HEPA differed between groups in subgroup analysis. Regarding the relationship between HEPA and HRQoL differing between subgroups, an individualized physical activity program is needed for effective improvement of HRQoL in non-dialysis CKD patients.

We hypothesized the probable mechanism of the impact of HEPA on HRQoL. Although there are many hypotheses, the identification of exact mechanism of HEPA’s influence on HRQoL is still challenging. Neuroendocrine dysfunction might induce lethargy and depressive mood in CKD, and adequate physical activity has an effect on the neuroendocrine system [33]. It could also improve social interaction, mood, stress, self-efficacy, and cardiovascular health [34,35]. HRQoL is a comprehensive surrogate marker of an individual’s health, and HEPA might improve HRQoL through various aspects of both physical and mental domains.

Our study has many strengths, including a prospective observational design, robust data collection, and a large study population. We also used robust statistical methods, including minimized omitted variable bias with multiple imputation methods and basic statistical assumptions to ensure unbiased and consistent results. These strengths make our analyses reliable. Despite many strengths, our study also has a few limitations. First, we could not explain the exact causal relationship between HEPA and HRQoL as seen in all observation studies. However, observational studies are powerful tools to assess the epidemiologic relationships, and we capitalized on complimentary analytic methods for a robust evaluation of the effect of HEPA and relevant clinical outcomes [36]. Second, the interpretation of HRQoL is complex and difficult. Universally standard tools for evaluating the HRQoL are limited. Third, self-reported questionnaires might not be objective. Fourth, despite the wide range of risk adjustments, the risk of hidden bias, confounders, and omitted variables cannot be solved completely.

## 5. Conclusions

Our findings emphasize the importance of HEPA associated with HRQoL in patients with non-dialysis CKD. HEPA recommended in the CKD population could improve the patients’ HRQoL and might improve their health and disease prognosis. A further follow-up study is required to determine the recommended amount of individualized physical activity for patients with non-dialysis CKD.

## Figures and Tables

**Table 1:** Clinical characteristics of the study population.

Variables	Total Subjects (n = 1618)	Health-Enhancing Physical Activity		*p*-Value
		No (906)	Yes (712)	
Age (years)	52.3 ± 12.3	52.8 ± 12.4	51.7 ± 12.1	0.085
Female	605 (37.4%)	399 (44.0%)	206 (28.9%)	<0.001
Income				0.002
Low	405 (25.0%)	205 (22.6%)	200 (28.1%)	
Middle	847 (52.3%)	470 (51.9%)	377 (52.9%)	
High	366 (22.6%)	231 (25.5%)	135 (19.0%)	
Educational status				0.002
Below elementary school	169 (10.4%)	117 (12.9%)	52 (7.3%)	
Middle school	162 (10.0%)	93 (10.3%)	69 (9.7%)	
High school	561 (34.7%)	310 (34.2%)	251 (35.3%)	
Above university	726 (44.9%)	386 (42.6%)	340 (47.8%)	
Marital status				0.009
Married	1339 (82.8%)	748 (82.6%)	591 (83.0%)	
Unmarried	182 (11.2%)	91 (10.0%)	91 (12.8%)	
Divorced or windowed	97 (6.0%)	67 (7.4%)	30 (4.2%)	
Employed	992 (61.3%)	512 (56.5%)	480 (67.4%)	<0.001
Smoking history				<0.001
Current	823 (50.9%)	498 (55.0%)	325 (45.6%)	
Never	273 (16.9%)	157 (17.3%)	116 (16.3%)	
Ex-smoker	522 (32.3%)	251 (27.7%)	271 (38.1%)	
Diabetes mellitus	509 (31.5%)	310 (34.2%)	199 (27.9%)	0.021
Hypertension	1548 (95.7%)	856 (94.5%)	692 (97.2%)	0.011
Charlson comorbidity index	3.0 [2.0; 5.0]	3.0 [2.0; 5.0]	3.0 [2.0; 4.0]	0.01
Body mass index (kg/m^2^)	24.5 ± 3.4	24.4 ± 3.5	24.6 ± 3.3	0.239
Waist-hip ratio	0.9 ± 0.1	0.9 ± 0.1	0.9 ± 0.1	0.179
Hemoglobin (g/dL)	12.9 ± 2.0	12.7 ± 2.0	13.2 ± 2.0	<0.001
Serum albumin (g/dL)	4.2 ± 0.4	4.2 ± 0.5	4.2 ± 0.4	0.054
Serum uric acid (mg/dL)	7.0 ± 1.9	7.0 ± 1.9	7.0 ± 1.8	0.732
Fasting glucose (mg/dL)	99.0 [91.0; 111.0]	99.0 [91.0; 112.0]	98.5 [92.0; 110.5]	0.807
C-reactive protein	0.6 [0.2; 1.6]	0.6 [0.3; 1.6]	0.6 [0.2; 1.6]	0.6
Calcium (mg/dL)	9.1 ± 0.5	9.1 ± 0.6	9.2 ± 0.5	0.013
Phosphate (mg/dL)	3.7 ± 0.7	3.7 ± 0.7	3.6 ± 0.6	<0.001
Estimated glomerular filtration rate (mL/min/1.73 m^2^)	48.0 [29.1; 76.3]	44.2 [27.0; 73.1]	51.5 [32.1; 77.1]	0.004
CKD stages				0.002
1	280 (17.3%)	156 (17.2%)	124 (17.4%)	
2	306 (18.9%)	161 (17.8%)	145 (20.4%)	
3a	273 (16.9%)	130 (14.3%)	143 (20.1%)	
3b	339 (21.0%)	192 (21.2%)	147 (20.6%)	
4	320 (19.8%)	203 (22.4%)	117 (16.4%)	
5	100 (6.2%)	64 (7.1%)	36 (5.1%)	
Protein to creatinine ratio (g/g Creatinine)	0.5 [0.1; 1.5]	0.5 [0.1; 1.6]	0.5 [0.1; 1.4]	0.177
High density lipoprotein (mg/dL)	47.0 [38.0; 58.0]	47.0 [38.0; 57.0]	47.0 [39.0; 58.0]	0.377
Low density lipoprotein (mg/dL)	93.0 [73.0; 115.0]	95.5 [75.0; 118.0]	91.0 [71.5; 112.0]	0.002
Triglyceride (mg/dL)	134.0 [94.0; 195.0]	136.0 [95.0; 198.0]	130.0 [92.5; 190.0]	0.38
Mean ankle brachial pressure index	1.1 ± 0.1	1.1 ± 0.1	1.2 ± 0.1	0.131
Bone mineral density (total spine, g/cm^2^)	−0.1 [−1.0; 0.9]	−0.2 [−1.2; 0.7]	0.2 [−0.8; 1.1]	<0.001
Physical component summary score	73.5 ± 17.9	70.2 ± 18.7	77.7 ± 15.7	<0.001
Mental component summary score	70.5 ± 18.0	67.6 ± 18.5	74.1 ± 16.6	<0.001
Kidney disease component summary score	73.2 ± 12.8	71.1 ± 12.9	76.0 ± 12.2	<0.001

Significant at *p*-value < 0.05.

**Table 2:** Adjusted linear regression for health-enhancing physical activity and health-related quality of life.

	Beta Coefficients	Confidence Interval	*p*-Value
Kidney disease component summary score	2.56	1.496–3.624	<0.001
Symptoms/problem lists	1.043	−0.173–2.258	0.093
Work status	2.108	−0.63–4.846	0.131
Sexual function	6.577	2.526–10.628	0.001
Physician encouragement	1.61	−0.37–3.59	0.111
Effects of kidney disease	1.812	0.521–3.103	0.006
Cognitive function	0.861	−0.406–2.127	0.183
Sleep	2.042	0.359–3.725	0.017
Patient satisfaction	1.929	−0.311–4.17	0.091
Burden of kidney disease	3.111	0.669–5.552	0.013
Quality of social interaction	2.046	0.418–3.675	0.014
Social support	5.022	2.731–7.313	<0.001
Physical component summary score	4.058	2.517–5.599	<0.001
Physical function	3.538	2.182–4.895	<0.001
Role-physical	4.634	1.674–7.593	0.002
Bodily pain	2.344	0.408–4.28	0.018
General health	5.717	3.695–7.739	<0.001
Mental component summary score	4.193	2.529–5.857	<0.001
Emotional well-being	4.045	2.353–5.736	<0.001
Role-emotional	2.846	−0.446–6.139	0.09
Social function	3.262	1.376–5.148	0.001
Energy/fatigue	6.62	4.803–8.437	<0.001

Significant at *p*-value < 0.05. Linear regression models were adjusted with age, sex, Charlson cormobidity index, systolic blood pressure, educational status, income status, work status, smoking history, marital status, waist-hip ratio, estimated glomerular filtration rate, low density lipoprotein, hemoglobin, mean ankle to brachial index, calcium, phosphate and bone mineral density.

## Data Availability

The datasets generated during and/or analyzed during the current study are available from the corresponding authors (S.K.M. and S.W.K.) on reasonable request.

## Supplementary Materials

The following supporting information can be downloaded at: https://www.mdpi.com/article/10.3390/ijerph19031318/s1, Table S1: Clinical characteristics of the study population before imputation, Table S2: Fully adjusted linear regression models.
