# Supplementary material for: The Association between Health-Enhancing Physical Activity and Quality of Life in Patients with Chronic Kidney Disease: Propensity Score Matching Analysis
Source: Int J Environ Res Public Health. 2022 Jan 25;19(3):1318. doi: 10.3390/ijerph19031318 (PMC8835055; doi:10.3390/ijerph19031318)
Supplement: Supplementary file 1 [file ijerph-19-01318-s001.zip › ijerph-1520849-supplementary.pdf]

The association between health-enhancing physical activity and quality of life in patients with chronic kidney disease: propensity score matching analysis

Tae Ryom Oh, M.D., Ph.D.[1], Hong Sang Choi, M.D., Ph.D.[1], Sang Heon Suh, M.D., Ph.D.[1], Chang Seong Kim, M.D., Ph.D.[1], Eun Hui Bae, M.D., Ph.D.[1], Suah Sung, M.D., Ph.D.[2], Seung Hyeok Han, M.D., Ph.D.[3], Kook Hwan Oh, M.D., Ph.D.[4], Seong Kwon Ma, M.D., Ph.D.[1,*], Soo Wan Kim, M.D., Ph.D.[1,*]

[1]Department of Internal Medicine, Chonnam National University Medical School and Chonnam National University Hospital, Gwangju, Korea; [2]Department of Internal Medicine, Eulji Medical Center, Eulji University, Seoul, Korea; [3]Department of Internal Medicine, College of Medicine, Institute of Kidney Disease Research, Yonsei University, Seoul, Korea; [4]Department of Internal Medicine, Seoul National University, Seoul, Korea

[*]These authors have contributed equally to this manuscript as correspondence authors.

Correspondence

*Soo Wan Kim, MD, PhD.

Department of Internal Medicine, Chonnam National University Medical School

42 Jebongro, Gwangju 61469, Korea

Tel: +82-62-220-6271

Fax: +82-62-225-8578

Email: skimw@chonnam.ac.kr

*Seong Kwon Ma, MD, PhD.

Department of Internal Medicine, Chonnam National University Medical School

42 Jebongro, Gwangju 61469, Korea

Tel: +82-62-220-6579

Fax: +82-62-225-8578

Email: drmsk@hanmail.net

Table S1. Clinical characteristics of the study population before imputation

Variables	No. of missing values (%)	Total subjects (n = 1618)	Health-enhancing physical activity		p-value
			No (906)	Yes (712)	
Age (years)	0 (0)	52.3 ± 12.3	52.8 ± 12.4	51.7 ± 12.1	0.085
Female	0 (0)	605 (37.4%)	399 (44.0%)	206 (28.9%)	<0.001
Income	47 (2.905)				0.004
Low		399 (25.4%)	203 (23.1%)	196 (28.3%)	
Middle		825 (52.5%)	457 (52.1%)	368 (53.1%)	
High		347 (22.1%)	218 (24.8%)	129 (18.6%)	
Educational status	0 (0)				0.002
Below elementary school		169 (10.4%)	117 (12.9%)	52 (7.3%)	
Middle school		162 (10.0%)	93 (10.3%)	69 (9.7%)	
High school		561 (34.7%)	310 (34.2%)	251 (35.3%)	
Above university		726 (44.9%)	386 (42.6%)	340 (47.8%)	
Marital status	0 (0)				0.009
Married		1339 (82.8%)	748 (82.6%)	591 (83.0%)	
Unmarried		182 (11.2%)	91 (10.0%)	91 (12.8%)	
Divorced or windowed		97 (6.0%)	67 (7.4%)	30 (4.2%)	
Employed	0 (0)	992 (61.3%)	512 (56.5%)	480 (67.4%)	<0.001
Smoking history	0 (0)				< 0.001
Current		823 (50.9%)	498 (55.0%)	325 (45.6%)	
Never		273 (16.9%)	157 (17.3%)	116 (16.3%)	
Ex-smoker		522 (32.3%)	251 (27.7%)	271 (38.1%)	
Diabetes mellitus	0 (0)	509 (31.5%)	310 (34.2%)	199 (27.9%)	0.021
Hypertension	0 (0)	1548 (95.7%)	856 (94.5%)	692 (97.2%)	0.011
Charlson comorbidity index	0 (0)	3.0 [2.0; 5.0]	3.0 [2.0; 5.0]	3.0 [2.0; 4.0]	0.01
Body mass index (kg/m^2)	9 (0.556)	24.5 ± 3.4	24.4 ± 3.5	24.6 ± 3.3	0.264
Waist-hip ratio	78 (4.821)	0.9 ± 0.1	0.9 ± 0.1	0.9 ± 0.1	0.201
Hemoglobin (g/dL)	17 (1.051)	12.9 ± 2.0	12.7 ± 2.0	13.2 ± 2.0	<0.001
Serum albumin (g/dL)	7 (0.433)	4.2 ± 0.4	4.2 ± 0.5	4.2 ± 0.4	0.057
Serum uric acid (mg/dL)	10 (0.618)	7.0 ± 1.9	7.0 ± 1.9	7.0 ± 1.8	0.753

Fasting glucose (mg/dL)	13 (0.803)	99.0 [91.0;112.0]	98.0 [92.0;110.0]	99.0 [91.0;111.0]	0.74
C-reactive protein	113 (6.984)	0.6 [0.2; 1.6]	0.6 [0.2; 1.6]	0.6 [0.2; 1.6]	0.488
Calcium (mg/dL)	9 (0.556)	9.1 ± 0.5	9.1 ± 0.6	9.2 ± 0.5	0.013
Phosphate (mg/dL)	10 (0.618)	3.7 ± 0.7	3.7 ± 0.7	3.6 ± 0.6	<0.001
Estimated glomerular filtration rate) mL/min/1.73 m^2)	0 (0)	48.0 [29.1;76.3]	44.2 [27.0;73.1]	51.5 [32.1;77.1]	0.004
Protein to creatinine ratio (g/g Creatinine)	33 (2.04)	0.5 [0.1; 1.6]	0.5 [0.1; 1.4]	0.5 [0.1; 1.5]	0.16
High density lipoprotein (mg/dL)	27 (1.669)	47.0 [38.0;57.0]	47.0 [39.0;58.0]	47.0 [38.1;58.0]	0.399
Low density lipoprotein (mg/dL)	25 (1.545)	95.0 [75.0;118.0]	91.0 [72.0;112.0]	93.0 [73.0;115.0]	0.004
Triglyceride (mg/dL)	46 (2.843)	137.0 [95.0;198.0]	131.0 [93.0;192.0]	134.0 [94.0;196.5]	0.436
Mean ankle brachial pressure index	30 (1.854)	1.1 ± 0.1	1.1 ± 0.1	1.2 ± 0.1	0.172
Bone mineral density (total spine, g/cm^2)	0 (0)	-0.1 [-1.0; 0.9]	-0.2 [-1.2; 0.7]	0.2 [-0.8; 1.1]	<0.001
Physical component summary score	0 (0)	73.5 ± 17.9	70.2 ± 18.7	77.7 ± 15.7	<0.001
Mental component summary score	0 (0)	70.5 ± 18.0	67.6 ± 18.5	74.1 ± 16.6	<0.001
Kidney disease component summary score	0 (0)	73.2 ± 12.8	71.1 ± 12.9	76.0 ± 12.2	<0.001

Significant at p-value < 0.05.

Table S2. Fully adjusted linear regression models

A) Kidney disease component summary score

	Beta coefficients	Confidence interval	p-value
Intercept	65.535	50.584 – 80.486	<0.001
Health enhancing physical activity	2.56	1.496 – 3.624	<0.001
Age	0.085	0.012 – 0.159	0.023
Male	1.982	0.282 – 3.682	0.022
Charlson comorbidity index	-1.225	-1.663 – -0.786	<0.001
Systolic blood pressure	0.02	-0.014 – 0.053	0.246
Education: M	1.558	-0.722 – 3.838	0.18
Education: H	3.777	1.845 – 5.708	<0.001
Education: U	3.059	1.023 – 5.095	0.003
Income: M	-0.475	-1.765 – 0.815	0.47
Income: H	-3.705	-5.4 – -2.011	<0.001
Unemployed	-5.921	-7.139 – -4.703	<0.001
Smoking: Never	-2.815	-4.526 – -1.105	0.001
Smoking: Ex	-1.742	-3.234 – -0.251	0.022
Married: Never	-7.068	-9.067 – -5.069	<0.001
Married: Ex	-3.05	-5.271 – -0.829	0.007
Waist-hip ratio	-4.596	-13.647 – 4.454	0.319
Estimated glomerular filtration rate	-0.007	-0.032 – 0.017	0.546
Low density lipoprotein	-0.009	-0.027 – 0.008	0.287
Hemoglobin	1.026	0.658 – 1.395	<0.001
Mean ankle brachial pressure index	4.621	-0.903 – 10.144	0.101
Calcium	0.002	-1.049 – 1.053	0.998
Phosphate	-2.259	-3.139 – -1.38	<0.001
Bone mineral density (total spine)	0.141	-0.227 – 0.509	0.453

significant at p-value < 0.05.

B) Physical component summary score

	**Beta coefficients**	**Confidence interval**	**p-value**
Intercept	83.11	61.448 – 104.772	<0.001
Health enhancing physical activity	4.058	2.517 – 5.599	<0.001
Age	0.128	0.022 – 0.235	0.018
Male	4.626	2.163 – 7.088	<0.001
Charlson comorbidity index	-1.691	-2.326 – -1.055	<0.001
Systolic blood pressure	-0.004	-0.053 – 0.044	0.868
Education: M	3.372	0.069 – 6.675	0.045
Education: H	7.272	4.473 – 10.07	<0.001
Education: U	7.671	4.721 – 10.621	<0.001
Income: M	-0.978	-2.847 – 0.891	0.305
Income: H	-6.918	-9.373 – -4.464	<0.001
Unemployed	-4.357	-6.122 – -2.593	<0.001
Smoking: Never	-3.769	-6.247 – -1.29	0.003
Smoking: Ex	-1.405	-3.566 – 0.756	0.202
Married: Never	-1.559	-4.455 – 1.337	0.291
Married: Ex	-1.573	-4.791 – 1.645	0.338
Waist-hip ratio	-23.217	-36.33 – -10.104	0.001
Estimated glomerular filtration rate	-0.003	-0.038 – 0.032	0.873
Low density lipoprotein	-0.014	-0.039 – 0.011	0.265
Hemoglobin	1.132	0.597 – 1.666	<0.001
Mean ankle brachial pressure index	4.042	-3.961 – 12.045	0.322
Calcium	-0.585	-2.108 – 0.938	0.451
Phosphate	-2.099	-3.374 – -0.825	0.001
Bone mineral density (total spine)	0.388	-0.145 – 0.922	0.154

C) Mental component summary score

	Beta coefficients	Confidence interval	p-value
Intercept	61.133	37.741 – 84.524	<0.001
Health enhancing physical activity	4.193	2.529 – 5.857	<0.001
Age	0.086	-0.029 – 0.201	0.143
Male	1.334	-1.325 – 3.993	0.325
Charlson comorbidity index	-0.793	-1.479 – -0.106	0.024
Systolic blood pressure	-0.008	-0.061 – 0.044	0.763
Education: M	3.653	0.086 – 7.22	0.045
Education: H	7.611	4.589 – 10.634	<0.001
Education: U	7.089	3.903 – 10.274	<0.001
Income: M	-0.134	-2.152 – 1.884	0.897
Income: H	-5.696	-8.347 – -3.046	<0.001
Unemployed	-2.134	-4.04 – -0.229	0.028
Smoking: Never	-4.599	-7.275 – -1.922	0.001
Smoking: Ex	-2.48	-4.814 – -0.147	0.037
Married: Never	-5.379	-8.506 – -2.252	0.001
Married: Ex	-1.127	-4.603 – 2.348	0.525
Waist hip ratio	-2.928	-17.088 – 11.232	0.685
Estimated glomerular filtration rate	-0.01	-0.048 – 0.028	0.598
Low density lipoprotein	-0.01	-0.037 – 0.017	0.465
Hemoglobin	1.286	0.709 – 1.863	<0.001
Mean ankle brachial pressure index	6.946	-1.696 – 15.588	0.115
Calcium	-0.601	-2.245 – 1.043	0.474
Phosphate	-2.967	-4.343 – -1.591	<0.001
Bone mineral density (total spine)	0.402	-0.174 – 0.978	0.172

Significant at p-value < 0.05. *Abbreviations: E in Education, below elementary school; M in Education, middle school; H in Education, high school; U in Education, above university; E in Work, employed; UE in Work, unemployed; WHR, waist-hip ratio; eGFR, estimated glomerular filtration rate; LDL, low density lipoprotein; ABI, ankle brachial pressure index; BMD, bone mineral density*
